# Metastasis of Pancreatic Intraductal Oncocytic Papillary Neoplasm to the Ovaries

**DOI:** 10.1155/crgm/9926744

**Published:** 2026-09-19

**Authors:** Emily Fronk, Jamie McDowell, Natalia Yanchenko, Hani Katerji, Ashlee Smith

**Affiliations:** ^1^ Department of Obstetrics and Gynecology, University of Rochester Medical Center, 601 Elmwood Avenue Box 668, Rochester 14642, New York, USA, rochester.edu; ^2^ Department of Obstetrics and Gynecology, Division of Gynecology Oncology, University of Rochester Medical Center, 601 Elmwood Avenue, Rochester 14642, New York, USA, rochester.edu; ^3^ Department of Pathology and Laboratory Medicine, University of Rochester Medical Center, 601 Elmwood Avenue, Rochester 14642, New York, USA, rochester.edu

## Abstract

**Objectives:**

Intraductal oncocytic papillary neoplasm (IOPN) is a digestive system tumor typically found in the head of the pancreas. Most often, they remain confined to the pancreas; it is rare that they develop into an invasive carcinoma or metastasize.

**Methods:**

This paper presents the case of a 63‐year‐old postmenopausal female with a past medical history notable for BRCA1 mutation and exploratory laparotomy with pancreatoduodenectomy who underwent risk‐reducing laparoscopic bilateral salpingo‐oophorectomy.

**Results:**

Postoperative pathology revealed metastatic oncocytic papillary neoplasm involving the bilateral ovaries. She was ultimately referred to gastrointestinal medical oncology for further management.

**Conclusions:**

This is the first report to our knowledge of pancreatic IOPN metastasizing to the ovaries. It is essential for cases to be reported for guidance of treatment regimens.

## 1. Introduction

First described in 1996, intraductal oncocytic papillary neoplasm (IOPN) is a rare disease accounting for approximately 4.5% of pancreatic intraductal neoplasms [1, 2]. In this case report, we describe a patient with a germline pathogenic variant of BRIP1 and history of an IOPN, found to have metastatic deposits of IOPN in bilateral ovaries. To our knowledge, this is the first report of this type of pancreatic lesion metastasizing to the ovaries.

## 2. Case Report

**FIGURE 1:**
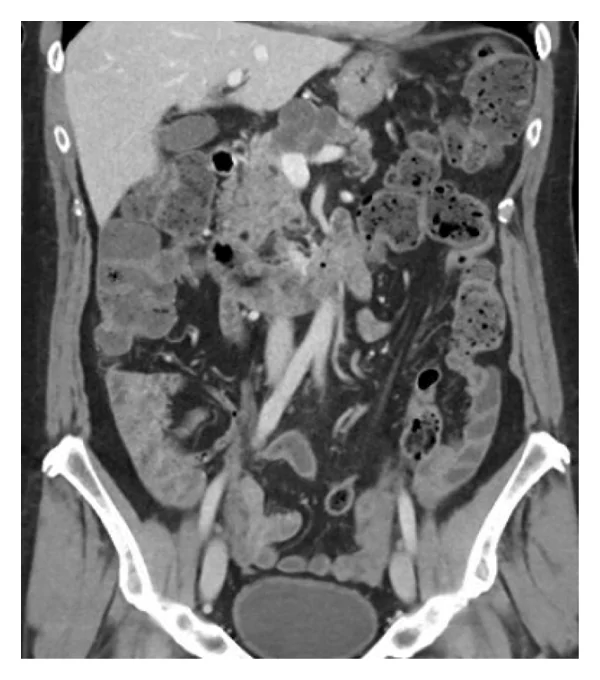
The patient’s CT in the coronal plane with a 4.9 × 4.7 cm complex, septated cystic pancreatic mass.

**FIGURE 2:**
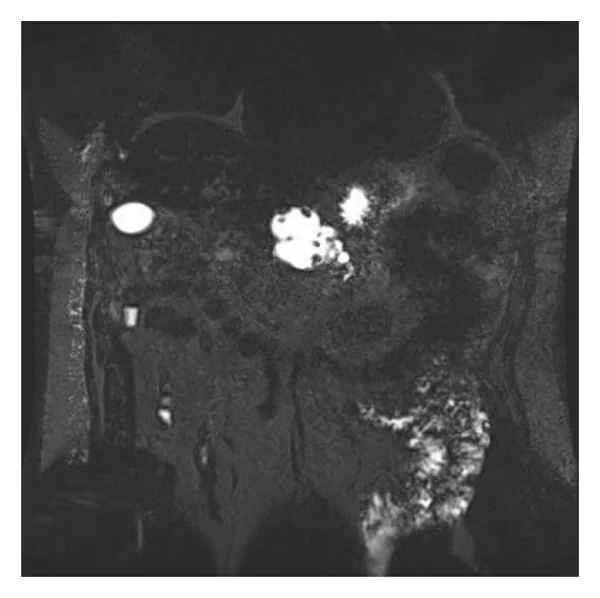
The patient’s MRI in the coronal plane with a 5.0 × 3.9 × 3.8 cm cystic mass with multiple enhancing mural nodules.

**FIGURE 3:**
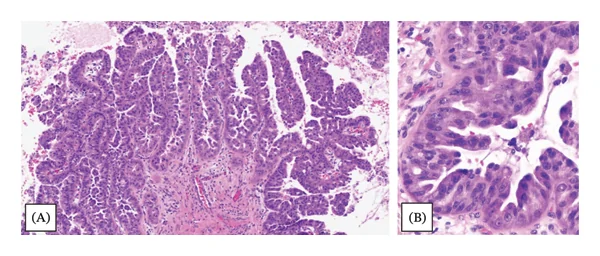
Patient’s initial pancreatic pathology. (A) Tumor in the pancreas, 10*x*, and (B) tumor in the pancreas, 40*x*.

In June 2023, a 63‐year‐old postmenopausal nulligravid patient presented to the gynecologic oncology clinic to discuss risk‐reducing surgery in the setting of a known deleterious BRCA1‐interacting protein C‐terminal helicase 1 (BRIP1) pathogenic variant. Her past medical history was otherwise notable for diagnosis of stage IA invasive ductal carcinoma of the right breast (ER positive, PR negative, and Her2 positive) in February 2023, subsequently treated with lumpectomy, radiation, and chemotherapy. After extensive counseling, she decided to undergo a risk‐reducing bilateral salpingo‐oophorectomy. Gynecologic surgery was ultimately postponed due to an incidentally found 4.9 × 4.7 cm complex, septated cystic pancreatic mass seen on a CT of the abdomen and pelvis in September 2023 (Figure 1). Subsequent abdominal MRI showed a 5.0 × 3.9 × 3.8‐cm cystic mass in the body of the pancreas with multiple enhancing mural nodules concerning for neoplasm (Figure 2). Laboratory testing revealed a CA 19–9 of 16 U/mL, CA 27.29 of 25 U/mL, and CA 15–3 of 17 U/mL. Due to concern for a precancerous or cancerous pancreatic lesion, she was taken to the operating room in November 2023 for an exploratory laparotomy with pancreatoduodenectomy with proximally extended subtotal pancreatectomy. Final pathology demonstrated an IOPN (Figures 3A,B). The pancreatic tail margin was positive for IOPN, and all remaining margins were negative. The small bowel, stomach, and gallbladder had no significant histologic abnormalities; all 14 lymph nodes were benign.

She returned to Gynecologic Oncology clinic in March 2025. Preoperative pelvic ultrasound was unremarkable with a normal appearing uterus and bilateral ovaries. Additionally, tumor markers were normal, with a CA‐125 of 7.4 and HE4 of 52.

**FIGURE 4:**
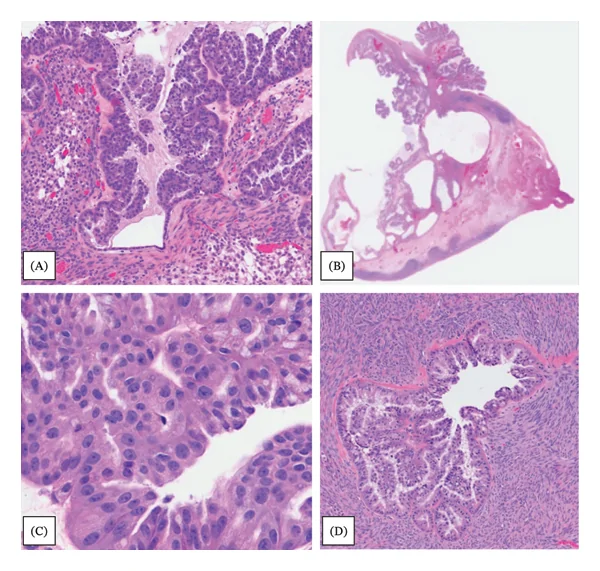
Patient’s ovarian pathology. (A) Tumor in the larger ovary, 10*x*; (B) tumor in the larger ovary, surface involvement and stromal invasion; (C) tumor in the larger ovary, 40*x*; and (D) tumor in the smaller ovary, 10*x*.

In April 2025, she underwent an uncomplicated laparoscopic bilateral salpingo‐oophorectomy and pelvic washings. Intraoperative findings were notable for a three‐cm simple cyst of the left ovary, and right ovary and bilateral fallopian tubes were unremarkable. Postoperative pathology revealed metastatic oncocytic papillary neoplasm involving the bilateral ovaries and adenofibromas of the bilateral fallopian tubes (Figures 4A–D). Pelvic washings were negative for malignancy.

Her case was reviewed by gynecologic oncology, radiation oncology, pathology, radiology, and hematology oncology at a multidisciplinary tumor board. Confirmatory secondary pathology review was also performed at another institution. The tumor board recommendation was for PET‐CT and referral to gastrointestinal medical oncology.

## 3. Discussion

In 2019, the World Health Organization classified IOPNs as a distinct type of rare digestive system tumor [3]. Though previously viewed as a subtype of intraductal papillary mucinous neoplasms (IPMNs), they have since been shown to have a different pathway of pathogenesis and minimal mucin production [3–5]. The majority of IOPNs are found in the head of the pancreas; however, this may be due to the tendency of pancreatic head lesions to present symptomatically [5]. Radiographically, they typically present with both solid and cystic mass components, leading to imaging similarities with pancreatic ductal adenocarcinoma, but they can be differentiated by the absence of KRAS mutations [3, 5]. Histologically, IOPNs appear as complex, branching papillae lined by oncocytic cells rich in mitochondria [5–7]. Only a minority of IOPNs develop into an invasive carcinoma, and most are confined to the pancreas; distant metastasis is rare [5, 8]. Due to its relatively indolent course, 5‐year survival is estimated to be between 90% and 95% [5, 6]. Adjuvant chemotherapy has not been shown to decrease disease recurrence in invasive IOPN, thus they are treated primarily by surgical resection [9].

BRIP1 is a Fanconi anemia protein involved in DNA interstrand crosslink repair [10, 11]. By interacting with the BRCT domain of BRCA1, as well as independent actions on cell cycle checkpoints, it acts to preserve the integrity of the genome. It is potentially associated with female breast cancer and strongly associated with epithelial ovarian cancer. Of patients with ovarian cancer, 0.9%–2.5% have been found to carry a defect [11, 12]. Additionally, those with a known BRIP1 pathogenic variant have a 5%–15% absolute risk for the development of ovarian cancer [12, 13]. There are insufficient data on whether BRIP1 mutations are associated with the development of pancreatic cancer [14].

Although there was no definitive evidence of invasion in this patient’s initial pancreatic pathology, surgical margins were positive at the pancreatic tail, raising concern for residual neoplasm in the remaining pancreas. Given the morphologic similarity of the lesion on her bilateral ovaries, it is most likely representative of dissemination from residual IOPN in the pancreas (Figure 2). It is, however, also possible that a second IOPN developed in her remaining pancreas and subsequently metastasized, that there were synchronous primary IOPNs, or that the initial IOPN was multicentric. However, after a multi‐institutional pathological review, the consensus remained that metastasis was the most likely explanation given the identical morphologies.

A literature review was taken to identify prevalence of and treatment options for IOPN lesions with metastasis to the ovaries. It was conducted via PubMed using the following terms: “intraductal oncocytic papillary neoplasm” and “ovarian metastasis.” One paper was identified but, upon further review, it did not report a case with this finding. Further searches were attempted with various term modifications, yet the authors were unable to find a paper describing this disease process. To our knowledge, this is the first report that describes a case of pancreatic IOPN with metastasis to the ovaries. There is a paucity of literature to guide treatment options for this patient’s disease.

## 4. Conclusion

IOPN is an intraductal pancreatic tumor that rarely evolves into invasive carcinoma. This case report describes a novel presentation of a patient with pancreatic IOPN metastasized to the ovaries. Due to the rarity, there is little information available to guide treatment; however, as more cases are reported, treatment regimens can be investigated.

## Author Contributions

Emily Fronk: conceptualization, writing–original draft, and writing–review and editing.

Jamie McDowell: conceptualization, writing–original draft, and writing–review and editing.

Natalia Yanchenko: conceptualization, writing–review and editing, and visualization.

Hani Katerji: conceptualization, writing–review and editing, and visualization.

Ashlee Smith: conceptualization, writing–review and editing, supervision, and project administration.

## Funding

No funding was received for this manuscript.

## Consent

Written informed consent was obtained from the patient for publication of this case report and accompanying images. A copy of the written consent is available for review on request.

## Conflicts of Interest

The authors declare no conflicts of interest.

## Data Availability

Data sharing not applicable to this article as no datasets were generated or analyzed during the current study.
